# Osteogenic Differentiation Modulates the Cytokine, Chemokine, and Growth Factor Profile of ASCs and SHED

**DOI:** 10.3390/ijms19051454

**Published:** 2018-05-14

**Authors:** Federico Mussano, Tullio Genova, Sara Petrillo, Ilaria Roato, Riccardo Ferracini, Luca Munaron

**Affiliations:** 1 CIR Dental School, Department of Surgical Sciences UNITO, via Nizza 230, 10126 Turin, Italy; tullio.genova@unito.it; 2 Department of Life Sciences and Systems Biology, UNITO, via Accademia Albertina 13, 10123 Turin, Italy; luca.munaron@unito.it; 3 Department of Molecular Biotechnology and Health Sciences, UNITO, Via Nizza 52, 10126 Turin, Italy; sara.petrillo@unito.it; 4 Center for Research and Medical Studies, A.O.U. Città della Salute e della Scienza, 10126 Turin, Italy; roato78@libero.it; 5 Department of Surgical Sciences (DISC), Orthopaedic Clinic-IRCCS A.O.U. San Martino, 16132 Genoa, Italy; riccardoferraciniweb@gmail.com

**Keywords:** mesenchymal stem cells (MSCs), adipose-derived stem cells (ASCs), stem cells from human exfoliated deciduous teeth (SHED), Bio-Plex

## Abstract

Great efforts have been made to improve bone regeneration techniques owing to a growing variety of sources of stem cells suitable for autologous transplants. Specifically, adipose-derived stem cells (ASCs) and stems cells from human exfoliated deciduous teeth (SHED) hold great potential for bone tissue engineering and cell therapy. After a preliminary characterization of the main biomolecules ASCs and SHED released in their conditioned media, cells were kept both in normal and osteo-inducing conditions. Conventional assays were performed to prove their osteogenic potential such as quantitative real-time polymerase chain reaction (qRT-PCR) (for RUNX-2, collagen type I, osteopontin and osteonectin), alkaline phosphatase activity, osteocalcin production, and von Kossa staining. Conditioned media were tested again after the osteogenic induction and compared to maintaining condition both at base line and after 14 days of culture. The osteogenic condition inhibited the release of all the biomolecules, with the exception, concerning SHED, of growth-regulated alpha protein precursor (GROα), and, to a lesser extent, interleukin (IL)-8. In conclusion, our data support that undifferentiated ASCs and SHED may be preferable to committed ones for general cell therapy approaches, due to their higher paracrine activity. Osteoinduction significantly affects the cytokine, chemokine, and growth factor profile in a differential way, as SHED kept a more pronounced pro-angiogenic signature than ASCs.

## 1. Introduction

With more than 2 million bone grafts performed annually worldwide, bone reconstruction is a primary task of regenerative medicine [1]. Autologous bone graft is deemed the safest and most effective grafting procedure [2]. However, the autograft entails a surgical “donor” site, which often brings additional morbidity, including pain and infections [3]. Moreover, the bone source is usually limited [4]. Xenografts, coming from other species, are hindered by the absence of cells and the possible contamination. Similarly, most available synthetic bone substitutes lack the ability to induce bone formation [5].

Whatever the type of grafting, the real challenge in bone tissue engineering is represented by critical-sized bone defect, meaning osseous injuries unable to heal spontaneously [6]. Only through an integrated approach will multidisciplinary teams succeed in fabricating complex scaffolds, including live cell populations endowed with the most sophisticated features of biomimicry [7], to set up a complex 3D tissue under the guidance of suitable biomolecular cues. To this end, the ideal stem cell should be abundant, accessible (i.e., harvested with a minimally invasive procedure), and capable of differentiating along multiple cell lineages in a reproducible manner [7,8].

Human mesenchymal stem cells (MSCs) have been shown to be particularly suitable for auto-transplant procedures. Apart from bone marrow [9], MSCs can be obtained from umbilical cord [10], adipose tissue [11], placenta [12,13], human synovial membrane [14], and dental pulp of deciduous teeth [15]. Teeth and the surrounding tissues are indeed an important source of MSCs. They can be categorized according to their origin in dental pulp stem cells (DPSCs), periodontal ligament stem cells, stem cells from apical pulp, dental follicular precursor cells, and stem cells from human exfoliated deciduous teeth (SHED) [16].

In the present study, the authors decided to focus on two easily attainable cell types, adipose-derived stem cells (ASCs) and SHED, as the most promising sources of MSCs in the dental field. Described for the first time in 2001 [17], ASCs, thus named as per the consensus of the International Fat Applied Technology Society [18], represent a plastic-adherent, multi-potent cell population that can be collected in large quantities with low possibility of donor site morbidity, during liposuction procedure [19]. SHED are highly proliferative, clonogenic cells capable of differentiating into a variety of cell types, including cell-mediating bone formation in vivo [20,21]. Owing to their short period of availability, limited to the primary dentition, SHED have been considered a prototypical source for banking [22].

The use of MSCs is mandatory for any bone engineering approach [23,24]. Besides their actual role in building new tissue, these cells are also attracting growing interest as “vehicles” for paracrine signals, in what is usually defined as cell therapy. Surprisingly, however, sporadic data are available thereof [25] and only minor information has been published comparing different mesenchymal cell types [26]. To the authors’ knowledge, indeed, no comparative studies have assessed the biomolecular profile characterizing ASCs and SHED so far, nor was the osteogenic capacity of ASCs and SHED ever tested under this perspective. Hence, the aim of the present study was to compare the cytokine, chemokine, and growth factor profile of osteoinduced ASCs and SHED to possibly unveil advantages and pitfalls of adopting either cell type in the context of regenerative medicine.

## 2. Results

### 2.1. ASCs and SHED Displayed Different Cell Morphology and Bimolecular Profiles at First Passage

**Figure 1:**
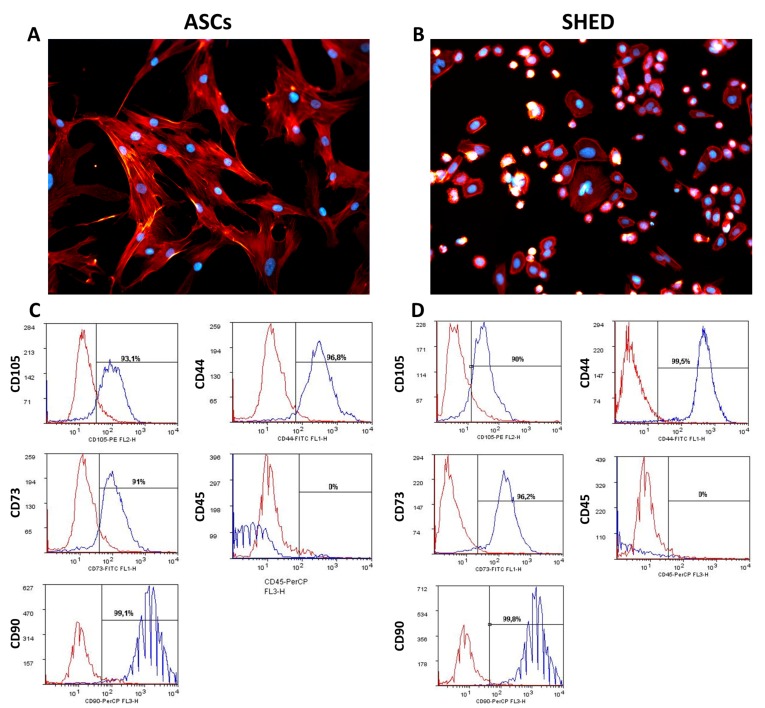
Cell morphology of adipose-derived stem cells (ASCs) (**A**) and stem cells from human exfoliated deciduous teeth (SHED) (**B**), phalloidin staining (in red) and DAPI (in blue) to mark respectively cytoskeleton and nuclei, magnification 400×. Cytofluorimetric characterization was obtained through CD105, CD44, CD73, CD90, and CD45 for ASCs (**C**), and SHED (**D**).

At the first in vitro cell culture passage after harvesting, ASCs appeared more spread out (Figure 1A), while SHED presented a higher nucleus to cytoplasm ratio (Figure 1B). ASCs and SHED showed a comparable phenotype consistent with that of MSCs [27]. Both cell types expressed the main mesenchymal markers CD105, CD44, CD73, and CD90 and were negative for CD45. No significant difference in the percentage expression of these markers was found in the two populations (Figure 1C,D).

**Figure 2:**
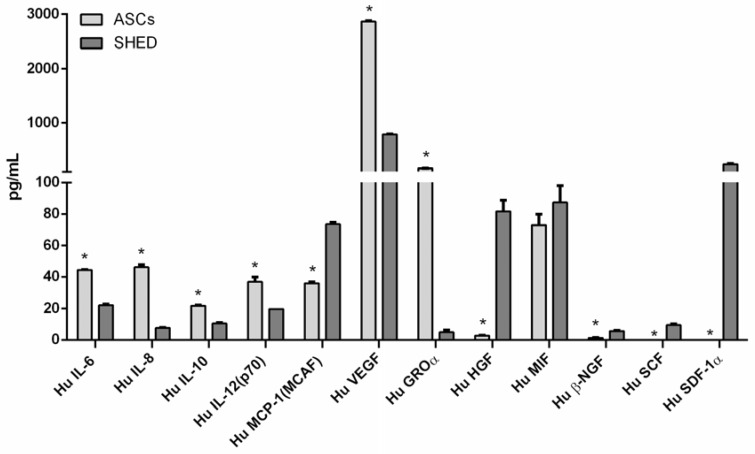
Bio-Plex quantification of factors produced by ASC and SHED at baseline. The evaluated factors are: interleukin-6 (IL-6), interleukin-8 (IL-8), interleukin-10 (IL-10), interleukin-12 (IL-12), monocyte chemoattractant protein-1 (MCP-1), vascular endothelial growth factor (VEGF), growth-regulated α protein precursor (GROα), hepatocyte growth factor (HGF), macrophage migration inhibitory factor (MIF), β-nerve growth factor (β-NGF), stem cell factor (SCF), and stromal cell-derived factor 1-α (SDF-1α). The symbol (*) indicates a significant difference between the two stem cells populations, considering a *p*-value < 0.05.

To assess the expression profile of growth factors, cytokines, and interleukins produced by these MSCs, a standard panel of factors was evaluated (Figure 2, Table 1). They include various anti- and pro-inflammatory interleukins (ILs), such as IL-6, -8, -10, and -12, as well as growth and angiogenic factors like growth-regulated alpha protein precursor (GROα) and vascular endothelial growth factor (VEGF), which were all significantly more abundant in ASCs than in SHED. On the contrary, while the levels of β-nerve growth factor (β-NGF), stem cell factor (SCF), and stromal cell-derived factor-1α (SDF-1α) were hardly detectable in ASCs, their production was significant in SHED, even in conspicuous quantities in the case of SDF-1α. Similarly, SHED released more monocyte chemotactic protein 1(MCP-1) and hepatocyte growth factor (HGF) (ten times higher levels) than ASCs. Macrophage migrator Inhibitory Factor (MIF) was well represented in both cell populations.

### 2.2. Cell Viability

**Figure 3:**
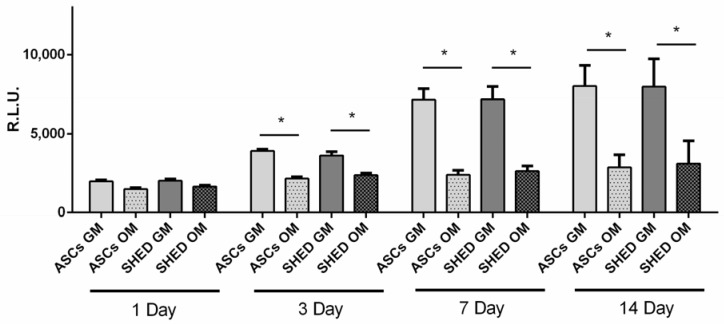
Cell viability assay. The assay was performed on ASCs and SHED cultured in growth medium (GM) or osteogenic medium (OM). Evaluations were made at 1, 3, 7, and 14 days from the beginning of the differentiation using CellTiter-Glo^®^ luminescent assay. Cell number is expressed as relative luminescence units (RLU). The symbol (*) indicates a significant difference between basal condition (GM) and osteogenic media (OM) considering a *p*-value < 0.05.

The growth of ASCs and SHED was estimated under maintenance (growth medium, GM) and osteogenic (osteogenic medium, OM) conditions at day 1, 3, and 7 (Figure 3). Starting from day 1, the proliferation rate was prevented in both ASCs and SHED grown in OM compared to GM. This trend became statistically significant at days 3 and 7 (Figure 3).

### 2.3. Early Osteogenic Differentiation of ASCs and SHED

**Figure 4:**
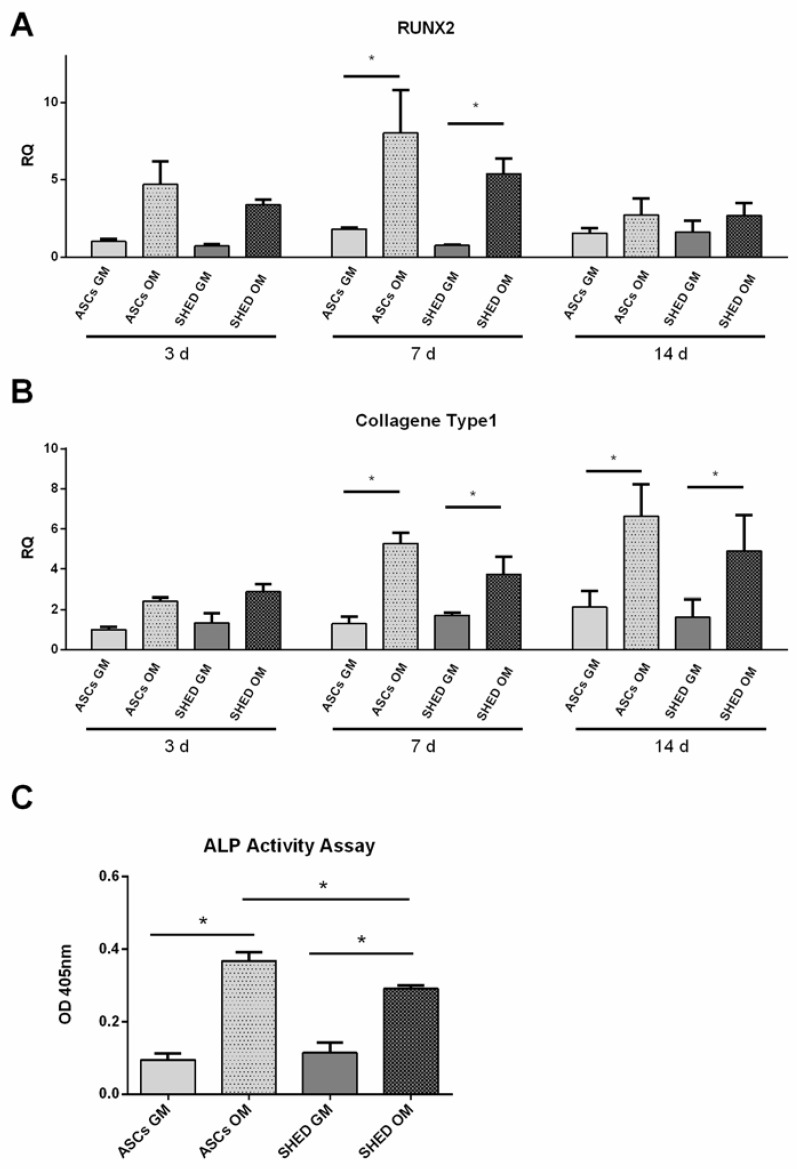
Early osteogenesis assessment based on quantitative real-time polymerase chain reaction (qRT-PCR) and alkaline phosphatase (ALP) activity assay. qRT-PCR analysis of osteogenic markers: RUNX2 (**A**), collagen type I (**B**), and ALP activity assay (**C**) performed on ASCs and SHED under basal conditions (GM) and in differentiating medium (OM). The symbol (*) indicates a significant difference between GM and OM condition considering a *p*-value < 0.05, d: day.

To assess the early osteogenic differentiation of ASCs and SHED, the transcript levels of RUNX-2 (Figure 4A) and collagen type I (Figure 4B) genes were evaluated through quantitative RT-PCR at days 3, 7, and 14. As a complementary assay to monitor the post-transcriptional level, alkaline phosphatase activity was also tested at day 7 (Figure 4C). These forms of evidence supported that both ASCs and SHED underwent proper osteogenic commitment, the bone markers being constantly higher in osteodifferentiated cells compared to the control (Figure 4). The statistical significance was achieved at day 7 in every assay.

### 2.4. Late Osteogenic Differentiation of ASCs and SHED

**Figure 5:**
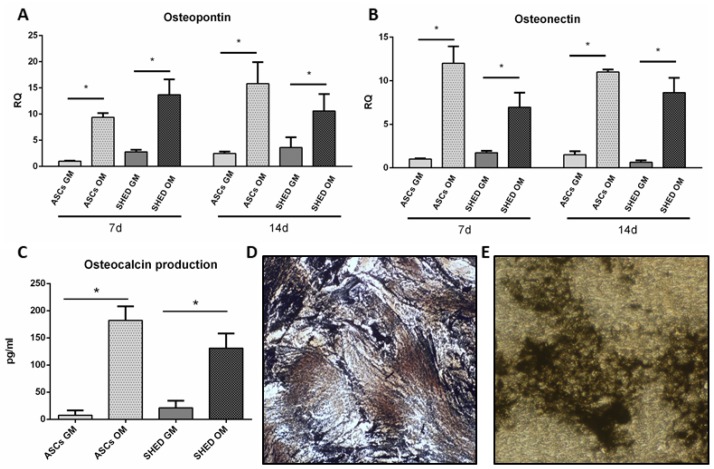
Late osteogenesis assessment based on qRT-PCR, ELISA detection of osteocalcin, and von Kossa staining. qRT-PCR analysis of osteopontin (**A**) and osteonectin (**B**) genes performed on ASCs (gray bar) and SHED (dark grey bar) under basal conditions (GM) and in osteo-differentiating medium (OM), d: day. Osteocalcin detection within conditioned media performed through ELISA (**C**). The symbol (*) indicates a significant difference between GM and OM condition considering a *p*-value < 0.05. Von Kossa Staining of osteodifferentiated ASCs (**D**) and SHED (**E**) at day 21, magnification 100×.

Late osteogenic differentiation of ASCs and SHED was evaluated by quantifying the transcriptional levels of osteopontin (Figure 5A) and osteonectin (Figure 5B) genes, which resulted in more expressed in OM condition at both day 7 and 14. Likewise, a significant enhancement of released osteocalcin was observed in osteoinduced cells compared to the controls at day 21 (Figure 5C). All these data clearly showed that ASCs and SHED were properly osteodifferentiated. As a further confirmation, the presence of calcified matrix was proven by von Kossa staining in osteoinduced ASCs and SHED at 21 days (respectively Figure 5D,E).

### 2.5. The Biomolecular Profile of ASCs and SHED Was Modulated upon Culture in OM

**Figure 6:**
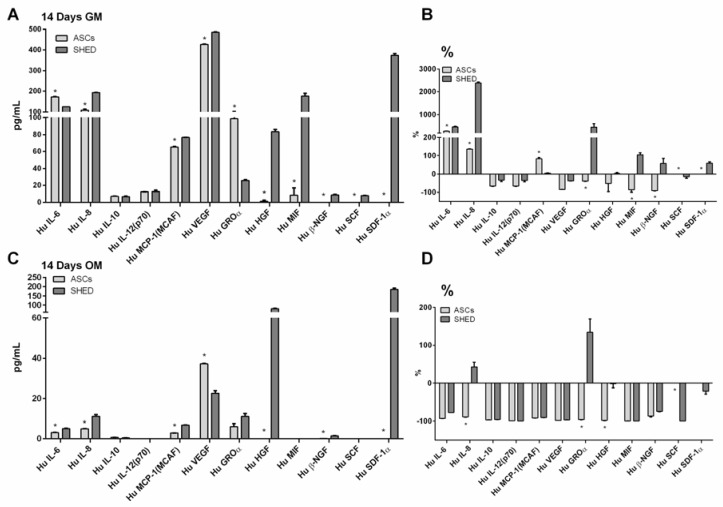
Bio-Plex quantification of factors produced by ASCs and SHED after 14 days of culturing in GM (**A**) and upon osteoinduction in OM (**C**). Side graphs comparing the relative variation of **A** and **C** (percentage) to the baseline after harvesting are shown respectively in (**B**) and (**D**) under the label %. The evaluated factors are: interleukin-6 (IL-6), interleukin-8 (IL-8), interleukin-10 (IL-10), interleukin-12 (IL-12), monocyte chemoattractant protein-1 (MCP-1), vascular endothelial growth factor (VEGF), growth-regulated alpha protein precursor (GROα), hepatocyte growth factor (HGF), macrophage migration inhibitory factor (MIF), β-nerve growth factor (β-NGF), stem cell factor (SCF), and stromal cell-derived factor 1-α (SDF-1α). All of the factors were analyzed in both populations, but b-NGF, SCF, and SDF-1α were not expressed in ASC. The symbol (*) indicates a significant difference between the two stem cell populations, considering a *p*-value < 0.05.

The expression profile of growth factors, cytokines, and interleukins produced by ASCs and SHED was again detected with the same panel that was evaluated after the harvest (Figure 2). After 14 days of culturing in GM (Figure 6A,B, Table 2), ASCs increased their levels of IL-6, IL-8, and MCP, while SHED released more IL-6, IL-8, GROα, MIF, and SDF1α. The comparison of the cells conditioned in OM for 14 days (Figure 6C,D, Table 2) with the baseline of freshly harvested cells revealed that the osteogenic condition inhibits the release of all the biomolecules expressed by ASCs and SHED, with the exception for the latter of GROα, and, to a lesser extent, IL-8.

## 3. Discussion

In recent years, great efforts have been made to improve bone regeneration techniques, owing not only to the technological innovations in the field of material science, but also to a growing variety of sources of stem cells for autologous transplants [8,28]. Among all the possible options, MSCs hold great potential for bone tissue engineering [29]. In the present work, we focused our attention on ASCs and SHED [17,20,21,30] respectively due to their abundance and their accepted bankability based on cryopreservation [31]. Interestingly, we showed that ASCs and SHED display distinctive morphologies when they adhere on plastic culture dishes, as it was portrayed even here (Figure 1A,B) through immunofluorescent staining. The standard panel of markers used [27] qualified both cell types as mesenchymal stem cells (Figure 1C,D).

Despite their common mesenchymal nature, ASCs and SHED derive from different tissues, and the microenvironment in which these MSCs live may deeply affect their gene expression profile [32] as well as their secretome. Thus, the purpose of this study was to evaluate and compare the effect of osteodifferentiation on the biomolecular expression profile of ASCs and SHED. To accomplish this task, a standard panel of growth factors, cytokines, and interleukins was evaluated (Figure 2), through Bio-Plex assay [25]. This technique has multiple advantages over common ELISA kits, since it permits a large number of molecules to be analyzed in the same sample and also allows reproducible and comparable titration curves to be established [33,34].

We found that ASCs and SHED strongly differed in their basal expression of several biomolecules. Important factors like HGF and SDF-1α were abundant in SHED but barely detectable in ASCs. Cloned as a mitogenic protein for hepatocytes [35], HGF promotes morphogenesis and cell survival. HGF was also proven to induce epithelial tubulogenesis/morphogenesis [36] and it has become a promising candidate for treating patients with impaired tissue function [37]. As for SDF-1α, it is a strong chemoattractant for stem cells that has received growing attention as a means to recruit endogenous progenitor cells possibly leading to regeneration in situ [38]. SCF, the ligand of the tyrosine kinase receptor c-KIT, and β-NGF were more expressed by SHED than ASCs, but did not reach high levels. These biomolecules might sustain trophic mechanisms and cooperate to allow for the successful use of MSCs for musculoskeletal applications [39]. The pro-inflammatory cytokine MIF was almost equally produced in both cell types. ASCs released higher levels of multifunctional interleukins (IL-6, IL-8, IL-10, IL-12) and VEGF, the prototypical pro-angiogenic factor, than SHED, whilst GROα was scarce in SHED, but fairly represented in ASCs.

Surprisingly, very little information is available in the literature concerning secretome of ASCs and SHED; indeed, it is quite rare to find reports where more than five cytokines/growth factors are analyzed at the same time. Different works reported the release of high amounts of VEGF by ASCs [40,41,42], similar to our result. Park et al. [43] examined the ASC secretion profile and its potential usage in treating skin ageing. Moreover, through the usage of enzyme-linked immunosorbent assay kits, these authors quantified HGF, which, in discord with our observation, resulted in the range of 670 pg/mL. The conspicuous production of HGF in SHED was instead described by Yamaguchi S et al. as was the low amount released by ASCs used in comparison [26], results which are pretty consistently in line with the outcome of our study (Figure 2). 

Osteogenic condition inhibited cell proliferation, as it could be easily predicted, in accordance with previous literature [44,45]. Likewise, the efficacy of the osteo-inductive condition adopted was assessed with standard methods, which consistently confirmed that the in vitro differentiation sought occurred properly. Interestingly, we found that ASCs increased their levels of IL-6, IL-8, and MCP, while SHED augmented the release of IL-6, IL-8, GROα, MIF, and SDF1α when cells became confluent, as it was clearly portrayed in the Bio-Plex analysis at 14 days (Figure 6A). 

The secretion of paracrine factors has been pointed out as a major way to enhance the healing process in numerous approaches based on ASCs [46,47] and SHED [48,49]. Based on these forms of evidence, the effect elicited by osteoinduction on the biomolecular profile of ASCs and SHED becomes of great interest. Osteogenic commitment tended to considerably downregulate the paracrine activity and thus the release of notably useful factors when compared to the baseline condition (shortly after cell harvest). Quite relevantly, all the biomolecules decreased, with the only exception of GROα and IL-8 in osteoinduced SHED. GROα, also known as CXCL1, was described among the key angiogenic chemokines [50], while the pro-angiogenic role of IL-8 has been acknowledged since the paramount study by Koch and colleagues [51]. Owing to this pro-angiogenic cue, committed SHED could prove useful to implement ex vivo bone regenerative Good Manufacturing Practice compliant protocols, especially in the dental field.

## 4. Materials and Methods

### 4.1. Primary Cell Harvest and Culture

The primary human cells used in this study, adipose-derived stem cells (ASCs) and stem cells from human exfoliated eciduous teeth (SHED), were obtained from human volunteers between 1 January and 30 June 2013. The research was conducted following the protocols approved (in October 2012) by the Ethics Committee of the Clinica Fornaca di Sessant (usage of lipoaspiration product, protocol number EC20121008) and the CIR-Dental School of the University of Turin (harvest of deciduous teeth, protocol number CIR20121022). Written informed consent was always obtained from donors. As previously reported [17], ASCs were isolated and expanded from the lipoaspirates of ten different donors (mean age 25.6 ± 4.5 years, male/female = 5/5) at the Clinica Fornaca di Sessant, Turin. Following Miura’s protocol [20], SHED were extracted from integer exfoliated deciduous teeth that were collected from ten children (9.2 ± 2.2 years) undergoing tooth extraction at the CIR-Dental School. For both cell types, the non-adherent cell populations were removed after 48 h and the adherent cell layer was washed twice with fresh medium. ASCs and SHED were maintained in growth medium (GM) consisting of RPMI (EuroClone S.p.A., Milan, Italy) enriched with sodium pyruvate and supplemented with 10% fetal bovine serum (Gibco BRL, Milan, Italy), penicillin-streptomycin 1:100, at 37 °C in humidified atmosphere with 5% CO_2_. Cells were then continuously cultured following their harvest until fourth passage. To induce osteogenic differentiation, cells were cultured in “osteogenic medium” (OM) by supplementing GM with 10 mM ß-glycerophosphate, 50 μg/mL ascorbic acid, and 0.02 mg/mL dexamethasone [52,53].

### 4.2. Cell Morphology

As described elsewhere [54,55,56], cells were seeded at a concentration of 5 × 10^3^ cells/well in a 24-well plate for 1 day. After fixing in 4% paraformaldehyde in phosphate buffered saline (PBS), cells were stained with rhodamine phalloidin and DAPI (Life Technologies, Milan, Italy) to mark actin network and nuclei, respectively. Images were acquired with a Nikon Eclipse Ti-E microscope using different a Nikon Plan 20X/0,10 (Nikon Instruments, Amsterdam, The Netherlands).

### 4.3. Phenotype of ASCs and SHED

Cell surface markers of ASCs and SHED were analyzed by flow cytometry. In detail, cells were identified as CD105, CD44, CD73, and CD90 positive cells and negative for CD45 expression. Standard labelling protocol was performed with the following antibodies fluorochrome-conjugated and isotypic controls: human CD105 PE (Invitrogen, Camarillo, CA, USA), CD73 FITC (kindly provided by Prof. Malavasi, University of Turin), CD44 FITC, CD45 PerCP, IgG1 PE and IgG2a PerCP (Miltenyi Biotech, Bergisch Gladbach, Germany), and IgG1 FITC-conjugated (Immunostep). As further control, unstained cells were also examined. About 10^5^ events/sample were used for capture with CellQuest software. Data were analyzed with FlowLogic software (Miltenyi Biotech).

### 4.4. Detection of Interleukins, Chemokines, and Growth Factors by Bio-Plex System

To quantify a panel of major signaling molecules released within their medium, ASCs and SHED were kept in GM for 3 days until confluence to assess the baseline. Cells were also kept either in GM or in osteodifferentiating medium for 14 days. The conditioned media thus produced by ASC and SHED after 2 hour starvation in RPMI were analyzed with the flexible Bio-Plex system (Bio-Rad Laboratories, Hercules, CA, USA), following the manufacturer’s protocol, as described elsewhere [57,58]. At least two independent experiments were made per sample. The concentrations of the following specific biomolecules was measured: IL-6, IL-8, IL-10, IL-12, MCP-1, VEGF, GROα, HGF, MIF, β-NGF, SCF, and SDF-1α. Concentrations of the analytes are expressed in pg/mL.

### 4.5. Viability Assay

ASCs and SHED were plated at a density of 500 cells/well in 96-well culture dishes and the viability was assessed by CellTiter-Glo^®^ (Promega, Milan, Italy) according to the manufacturer’s protocol at 1, 3, 7, and 14 days [59,60]. The CellTiter-Glo^®^ Luminescent Cell Viability Assay is a homogeneous method of determining the number of viable cells in culture based on quantitation of ATP as a marker of metabolically active cells. The amount of ATP is directly proportional to the number of viable cells in culture; for this reason, this assay can be used as an indicator of cell proliferation (as reported by manufacturer).

### 4.6. RNA Extraction and Real-time PCR Analysis

Total RNA was extracted using PureLink RNA Mini Kit (Ambion, Life Technologies Italy, Milan, Italy). For quantitative real-time polymerase chain reaction (qRT-PCR), 1 μg total RNA was transcribed into complementary DNA by MultiScribe^®^ Reverse Transcriptase (High-Capacity cDNA Reverse Transcription Kit, Thermo Fisher Scientific, Waltham, MA, USA) and PCR analysis was then assessed using TaqMan probes from Roche. Transcript abundance, normalized to 18s mRNA expression, is expressed as a fold increase over a calibrator sample. qRT-PCR was performed on a 7900HT Fast Real-Time PCR System (Applied Biosystems, Life Technologies Italy, Milan, Italy). Specific primers and probes were designed using the Universal Probe Library—Assay Design Center—Roche Life Science software (www.lifescience.roche.com) [61,62].

### 4.7. Alkaline Phosphatase Activity

Alkaline phosphatase activity (ALP) was determined colorimetrically as previously reported [63] and assessed at day 7. Cells were lysed with 0.05% Triton X-100 and incubated with the reagent solution containing phosphatase substrate (Sigma-Aldrich, Milan, Italy) at 37 °C for 15 min. A calibration curve of p-nitrophenol standards was always used. Alkaline phosphatase values were determined (Optical Density 405 nm) and normalized to the whole protein content, which was determined (Optical Density 562 nm) with a BCA™ Protein Assay (Thermo Fisher Scientific).

### 4.8. Osteocalcin Detection

Following the manufacturer’s protocol, osteocalcin (OCN) was measured in GM and OM media by means of Osteocalcin Elisa kit (KAQ1381 Invitrogen Corporation, Waltham, MA, USA) at day 21.

### 4.9. Von Kossa Staining

ASCs and SHED were grown in six-plate wells until day 21 in OM. Cells were washed once with PBS, then they were fixed with 4% paraformaldehyde for 10 min at room temperature prior to being washed again with PBS. Calcium salts were stained after von Kossa (vK) as reported previously [64], while representative pictures were captured under light microscopy by an Olympus camera.

### 4.10. Statistical Analysis

Data were analyzed by the use of GraphPad Prism6 (GraphPad Software, Inc., La Jolla, CA, USA) and Microsoft Excel (Microsoft Corporation, Redmond, WA, USA). Each experiment was repeated at least three times. Statistical analysis was performed by using the Mann-Whitney non-parametric *t*-test. A *p*-value of <0.05 was considered significant [65,66].

qRT-PCR data were analyzed with GraphPad Prism 6 (GraphPad Software, Inc., La Jolla, CA, USA) by performing ordinary two-way ANOVA with Sidak’s multiple comparisons test for grouped analyses or Mann Whitney test for column analyses.

## 5. Conclusions

In summary, these data suggest that undifferentiated ASCs and SHED may be preferable to committed ones for general cell therapy approaches, due to their higher paracrine activity. Osteoinduction significantly affects the cytokine, chemokine, and growth factor profile in a differential way, as SHED, differently from ASCs, developed a marked pro-angiogenic signature.

## Figures and Tables

**Table 1:** Bio-Plex quantification of factors produced by ASC and SHED at baseline. The evaluated factors are expressed in pg/mL and are: interleukin-6 (IL-6), interleukin-8 (IL-8), interleukin-10 (IL-10), interleukin-12 (IL-12), monocyte chemoattractant protein-1 (MCP-1), vascular endothelial growth factor (VEGF), growth-regulated alpha protein precursor (GROα), hepatocyte growth factor (HGF), macrophage migration inhibitory factor (MIF), β-nerve growth factor (β-NGF), stem cell factor (SCF), and stromal cell-derived factor 1-α (SDF-1α). Italic fonts of numbers indicate a significant difference between the two stem cell populations, considering a *p*-value < 0.05.

Biomolecule	Baseline	
	ASCs	SHED
Hu IL-6	*44.40*	*22.12*
Hu IL-8	*46.13*	*7.76*
Hu IL-10	*21.78*	*10.55*
Hu IL-12	*36.76*	*19.72*
Hu MCP-1	*35.82*	*73.40*
Hu VEGF	*2867.41*	*786.37*
Hu GROα	*161.90*	*4.90*
Hu HGF	*2.78*	*81.67*
Hu MIF	72.90	87.28
Hu β-NGF	*1.33*	*5.68*
Hu SCF	*0.01*	*9.51*
Hu SDF-1α	*0.01*	*233.43*

**Table 2:** Bio-Plex quantification of factors produced by ASCs and SHED after 14 days of culturing in GM and upon osteoinduction in OM. The evaluated factors are expressed in pg/mL and are: interleukin-6 (IL-6), interleukin-8 (IL-8), interleukin-10 (IL-10), interleukin-12 (IL-12), monocyte chemoattractant protein-1 (MCP-1), vascular endothelial growth factor (VEGF), growth-regulated alpha protein precursor (GROα), hepatocyte growth factor (HGF), macrophage migration inhibitory factor (MIF), β-nerve growth factor (β-NGF), stem cell factor (SCF), and stromal cell-derived factor 1-α (SDF-1α). Italic fonts of numbers indicate a significant difference between the two stem cell populations, considering a *p*-value < 0.05.

Biomolecule	14 Days GM		14 Days OM	
	ASCs	SHED	ASCs	SHED
Hu IL-6	*173.07*	*125.29*	*3.02*	*5.04*
Hu IL-8	*108.70*	*192.72*	*4.95*	*11.10*
Hu IL-10	7.24	6.95	0.62	0.39
Hu IL-12	12.41	12.73	0.01	0.01
Hu MCP-1	*65.23*	*76.71*	*2.87*	*6.80*
Hu VEGF	*426.58*	*486.12*	*37.25*	*22.67*
Hu GROα	*98.51*	*25.85*	5.99	11.03
Hu HGF	*1.43*	*83.51*	*0.10*	*79.66*
Hu MIF	*8.54*	*176.84*	0.10	0.00
Hu β-NGF	*0.10*	*8.87*	*0.17*	*1.45*
Hu SCF	*0.01*	*8.02*	0.01	0.10
Hu SDF-1α	*0.01*	*373.04*	*0.01*	*183.91*

## Abbreviations

MSCs	Mesenchymal stem cells
ASCs	Adipose-derived stem cells
SHED	Stem cells from human exfoliated deciduous teeth
DPSCs	Dental pulp stem cells
GM	Growth medium
OM	Osteogenic medium
VEGF	Vascular endothelial growth factor
FGFs	Fibroblast growth factors
IGF-1	Insulin growth factor 1
IL-6	Interleukin-6
IL-8	Interleukin-8
IL-10	Interleukin-10
IL-12	Interleukin-12
MCP-1	Monocyte chemoattractant protein-1
GROα	Growth-regulated alpha protein precursor
HGF	Hepatocyte growth factor
MIF	Macrophage migration inhibitory factor
β-NGF	β-Nerve growth factor
SCF	Stem cell factor
SDF-1α	Stromal cell-derived factor 1-α
qRT-PCR	Quantitative real-time polymerase chain reaction
PBS	Phosphate buffered saline
ALP	Alkaline phosphatase
OCN	Osteocalcin
